# Phylogenetic and structural analysis of annexins in pea (Pisum sativum L.) and their role in legume-rhizobial symbiosis development

**DOI:** 10.18699/VJ21.057

**Published:** 2021-09

**Authors:** O.A. Pavlova, I.V. Leppyanen, D.V. Kustova, A.D. Bovin, E.A. Dolgikh

**Affiliations:** All-Russia Research Institute for Agricultural Microbiology, Pushkin, St. Petersburg, Russia; All-Russia Research Institute for Agricultural Microbiology, Pushkin, St. Petersburg, Russia; All-Russia Research Institute for Agricultural Microbiology, Pushkin, St. Petersburg, Russia; All-Russia Research Institute for Agricultural Microbiology, Pushkin, St. Petersburg, Russia; All-Russia Research Institute for Agricultural Microbiology, Pushkin, St. Petersburg, Russia

**Keywords:** legume-rhizobial symbiosis, pea annexins, three-dimensional modeling, proteomics, calcium inhibitors, localization, бобово-ризобиальный симбиоз, аннексины гороха, 3D-моделирование, протеомика, ингибиторы кальция, локализация

## Abstract

Annexins as Ca2+/phospholipid-binding proteins are involved in the control of many biological processes essential for plant growth and development. In a previous study, we had shown, using a proteomic approach, that the synthesis of two annexins is induced in pea roots in response to rhizobial inoculation. In this study, phylogenetic analysis identif ied these annexins as PsAnn4 and PsAnn8 based on their homology with annexins from other legumes. The modeling approach allowed us to estimate the structural features of these annexins that might inf luence their functional activity. To verify the functions of these annexins, we performed comparative proteomic analysis, experiments with calcium inf lux inhibitors, and localization of labeled proteins. Essential down-regulation of PsAnn4 synthesis in a non-nodulating pea mutant P56 (sym10) suggests an involvement of this annexin in the rhizobial symbiosis. Quantitative RT-PCR analysis showed that PsAnn4 was upregulated at the early stages of symbiosis development, starting from 1–3 days after inoculation to up to 5 days after inoculation, while experiments with the Ca2+ channel blocker LaCl3 revealed its negative inf luence on this expression. To follow the PsAnn4 protein localization in plant cells, it was fused to the f luorophores such as red f luorescent protein (RFP) and yellow f luorescent protein (YFP) and expressed under the transcriptional regulation of the 35S promoter in Nicotiana benthamiana leaves by inf iltration with Agrobacterium tumefaciens. The localization of PsAnn4 in the cell wall or plasma membrane of plant cells may indicate its participation in membrane modif ication or ion transport. Our results suggest that PsAnn4 may play an important role during the early stages of pea-rhizobial symbiosis development.

## Abstract (ru)

Аннексины являются Ca2+/фосфолипид-связывающими белками, которые вовлечены в контроль многих биологических процессов, необходимых для роста и развития растений. Ранее выполненный протеомный анализ позволил нам выявить два аннексина, синтез которых усиливается в ответ на ризобиальную инокуляцию. В этой работе с помощью филогенетического анализа два аннексина были классифицированы как PsAnn4 и PsAnn8 на основании их гомологии с аннексинами других бобовых растений. С помощью молекулярного моделирования мы изучили структурные особенности этих аннексинов, которые могут влиять на их функциональную активность. Для анализа функции PsAnn4 и PsAnn8 были проведены сравнительный протеомный анализ, эксперименты с ингибиторами поступления кальция в клетку и локализация в тканях растений. Отсутствие активации синтеза PsAnn4 у мутанта гороха P56 (sym10), не способного формировать клубеньки, предполагает участие этого аннексина в бобово-ризобиальном симбиозе. Количественная ПЦР, совмещенная с обратной транскрипцией, показала, что экспрессия гена PsAnn4 увеличивается на ранних стадиях развития симбиоза начиная с 1–3-го дня после инокуляции до 5-го дня, тогда как блокатор Ca2+ канала LaCl3 подавляет эту экспрессию. Для изучения локализации PsAnn4 в клетках растений были получены конструкции для синтеза этого белка, слитого с такими флуорофорами, как красный флуоресцентный белок (RFP) и желтый флуоресцентный белок (YFP) при транскрипционной регуляции под промотором 35S в листьях Nicotiana benthamiana при инфильтрации Agrobacterium tumefaciens. Локализация PsAnn4 в клеточной стенке или плазматической мембране клеток растений указывает на возможность участия этого аннексина в ионном транспорте или модификации мембраны. Обсуждается возможная роль аннексина PsAnn4 в регуля- ции ранних стадий развития симбиоза у гороха.

## Introduction

Annexins are of particular research interest due to their ability to regulate various aspects of plant growth and development. Annexins belong to the evolutionarily conserved superfamily of proteins that are involved in Ca2+-dependent or Ca2+-independent binding with membrane phospholipids (Laohavisit, Davies, 2011; Davies, 2014). Most annexins have four putative annexin repeats of around 70 amino acids, with the conservative repeat GxGT-(38 residues)-D/E, which confers Ca2+/phospholipid-binding activity to these proteins (Gerke, Moss, 2002; Laohavisit, Davies, 2011). In addition, some plant annexins have motifs demonstrating F-actin binding and peroxidase and ATPase/GTPase activities (Mortimer et al., 2008; Konopka-Postupolska et al., 2011).

Despite the general structural similarity of these proteins, the functions of annexins are diverse, and individual annexins may have specific activities. Annexins are involved in a wide variety of essential cellular processes, including the regulation of membrane organization, vesicle trafficking, cytoskeletal dynamics, exocytosis, cell cycle control, ion transport, and signal transduction (Laohavisit, Davies, 2011; Clark et al., 2012; Davies, 2014). Annexins as phospholipid-binding proteins are being implicated in the fusion of membrane vesicles, as was shown for annexins from bell pepper and cotton (Clark et al., 2012; Lizarbe et al., 2013). They are also involved in the regulation of exocytosis, e. g., annexins in Zea mays root cap cells (Carroll et al., 1998). Moreover, annexins can function as cationic channels activated by various stimuli in cells. Annexins can influence the Ca2+ influx in plant cells, as was demonstrated for a Capsicum annuum annexin, which has Ca2+-channel activity (Hofmann et al., 2000). The Arabidopsis thaliana annexin AtAnn1, which is expressed in root cells, exhibits pH-dependent cation-channel activity, while Z. mays annexins cause active conductivity of Ca2+ in lipid bilayers at slightly acidic pH (Gorecka et al., 2005; Laohavisit et al., 2009). Since annexins can be Ca2+ sensors, these proteins are likely to be involved in signal transduction; for example, the annexin from Triticum aestivum was suggested to be engaged in low-temperature signaling (Breton et al., 2000).

Participation of annexins in the responses to cold, oxidative, and saline stresses is well-studied in plants (Mortimer et al., 2008; Clark et al., 2012; Espinoza et al., 2017). The annexin AtAnn1 from A. thaliana is involved in plant protection against oxidative stress (Konopka-Postupolska et al., 2009). The overexpression of AtAnn has been found to confer tolerance to drought and salt stresses and fungal attack in transgenic plants (Konopka-Postupolska et al., 2009). Similarly, the overexpression of the wild tomato (Solanum pennellii) annexin SpAnn2 in cultivated tomato Solanum lycopersicum enhances drought and salt tolerance through the elimination of reactive oxygen species (ROS) (Ijaz et al., 2017).

Some annexins are also known to be activated in plants during interaction with plant-growth promoting bacteria(Kwon et al., 2016) and the development of mutualistic symbioses (De Carvalho-Niebel et al., 1998, 2002; Wienkoop, Saalbach,2003; Manthey et al., 2004; Talukdar et al., 2009; Limpens et al., 2013; Breakspear et al., 2014; Carrasco- Castilla et al., 2018). During legume-rhizobial symbiosis, physiological changes occur, which are necessary for rhizobial infection and nodule organogenesis, such as the stimulation of ion fluxes, membrane depolarization, ROS production, cytoplasm alkalinization, perinuclear calcium oscillations, and cytoskeletal rearrangements. In Medicago truncatula, the transcription of MtAnn1 is activated directly by Nod factors or inoculation with rhizobia in epidermal cells and later in cortical cells (De Carvalho-Niebel et al., 1998, 2002; Breakspear et al., 2014). Studies using confocal microscopy showed GFP-labeled MtAnn1 to be localized in the cytoplasm, but protein accumulation in response to inoculation occurred at the periphery of the nucleus. MtAnn1 has been shown to be able to bind to the membrane phospholipid phosphatidylserine. Therefore, MtAnn1 is probably related to the events occurring at the early stages of symbiosis, leading to bacterial infection or nodule organogenesis (De Carvalho-Niebel et al., 2002).

Transcriptome profiling of roots inoculated with rhizobia revealed enhanced expression of MtAnn2, as well as MtAnn1 (Manthey et al., 2004). The expression of the MtAnn2 gene is associated with cell division in the nodule primordium (Manthey et al., 2004). Proteomic analysis revealed the MtAnn2 protein presence in lipid rafts from root plasma membrane preparations (Lefebvre et al., 2007). Another annexin MtAnn3 was found to be important for root hair deformations in M. truncatula (Gong et al., 2012). The increased expression of MtAnn1 and MtAnn2 is also associated with the early stages of AM fungal symbiosis, which corresponds to the stages of pre-infection and infection in this type of symbiosis (Manthey et al., 2004). This may indicate the general role of these annexins in the regulation of signaling pathways that lead to the development of two types of symbiosis.

A protein homologous to MtAnn1 – PvAnn1 from Phaseolus vulgaris – is activated at the early stages of symbiosis development (Jáuregui-Zúñiga et al., 2016; Carrasco-Castilla et al., 2018). The stimulation of Ca2+ ion transfer through the plasma membrane and ROS production caused by Nod factors constitute an early response in the signal transduction pathway. Analysis of PvAnn1-RNAi transgenic roots inoculated with rhizobia showed a decrease in ROS production and Ca2+ influx into the cells, which resulted in impaired progression and decreased numbers of infection threads and nodules (Carrasco-Castilla et al., 2018). Taken together, these findings point to the involvement of PvAnn1 in the regulation of signal transduction at early stages.

Previously performed proteomic analysis in pea (Pisum sativum L.) allowed us to reveal two annexins, the synthesis of which was increased in response to inoculation with Rhizobium leguminosarum bv. viciae RCAM1026 in 24 h (Leppyanen et al., 2018). In this work, searching in the recently released pea genome database using available coding sequences for annexin genes from M. truncatula and P. vulgaris revealed 15 annexins in pea. Phylogenetic analysis showed the relationship among members of the annexin superfamily in other legumes and allowed the identification of two previously revealed pea annexins responsive to rhizobial inoculation as PsAnn4 and PsAnn8 based on their homology with the M. truncatula and P. vulgaris proteins. To verify the function of these annexins, we performed comparative proteomic analysis using pea mutant P56 (sym10) unable to form symbiosis and wild type cv. Frisson. The approaches employed included quantitative RT-PCR, experiments with calcium channel inhibitors, and localization of labeled proteins.

## Materials and methods

Plant material and bacterial strain. Pea Pisum sativum L. seeds cv. Frisson were sterilized with sulphuric acid for 5 min, washed with water 3 times, transferred on 1 % water agar plates and germinated at room temperature in the dark. 4–5 days-old seedlings were transferred into pots with vermiculite saturated with Jensen medium (van Brussel et al., 1982), grown in a growth chamber at 21 °С at 16 h light/ 8 h dark cycles, 60 % humidity. For experiments with inhibitor, the Ca2+ channels blocker LaCl3, the plants were grown in pots saturated with Jensen medium with 100 μM CaCl2 × 2 H2O. The Rhizobium leguminosarum bv. viciae strain RCAM 1026 (WDCM 966) was cultivated at 28 °С on TY (Orosz et al., 1973) agar medium with 0.5 mg/ml of streptomycin. Fresh liquid bacterial culture was grown in B– medium (Van Brussel et al., 1977) and the optical density of the suspension at 600 nm (OD600) was adjusted to 0.5. Pea seedlings were inoculated with 2 ml of R. leguminosarum bv. viciae per plant. Pea roots (segments of main roots susceptible for rhizobial infection without lateral roots) were harvested 1 day after inoculation (dai).

Nicotiana benthamiana seeds were surface sterilized with 10 % hypochlorite for 10 min, washed with water 5 times and left for imbibition on a plate with sterile filter paper at 4 °С. All seeds were germinated in a large plastic box with soil for seven days, and then transferred into individual pots with soil. Plants were grown at 23 °С with 16 h light/8 h dark cycles, 60 % humidity.

Phylogenetic analysis. Multiple sequence alignments were performed using ClustalΩ http://www.clustal.org/omega/ (Sievers et al., 2011). The phylogenetic tree was generated with the Maximum Likelihood method using MEGA X https://www.megasoftware.net/ with 1000 bootstrap replicates. The domain composition of the corresponding encoded proteins was assessed using PFAM https://www.sanger.ac.uk/science/tools/pfam(Bateman et al., 2004).

Protein homology modeling was performed in Modeller 9.20 https://salilab.org/modeller/9.20/release.html(Webb, Sali, 2016). Visualization of the three-dimensional structure was obtained using the PyMol programhttps://pymol.org/2/ (Ordog, 2008). The three-dimensional crystal structure of the GhAnn1 G. hirsutum protein (Hu et al., 2008) was used as a template for building the model. To refine the model, the (VTFM) and the method of molecular dynamics in vacuum. The reliability of the model was calculated by the formula

**Formula 1.**
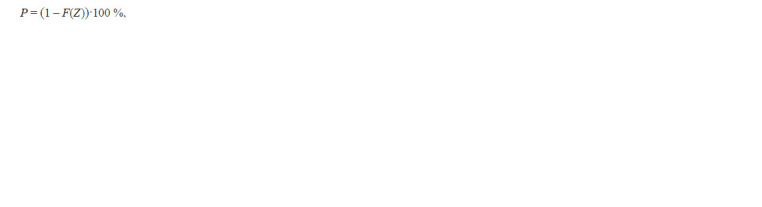
where Z is the estimation of discretely optimized protein energy, F is the Gaussian function with μ = 0 and σ2 = 1.

Isolation of total protein from pea roots. A modified method was used to isolate proteins from pea roots (Dam et al., 2014). 100 mg of the roots were ground in liquid nitrogen, then extraction buffer (0.1 M tris-HCl (pH 8.0), 30 % sucrose, 10 mM dithiothreitol (DTT), 2 % sodium dodecyl sulfate (SDS), a mixture of protease inhibitors (Sigma-Aldrich, USA) was added to the material and extraction was performed at +4 °С. After centrifugation at 12 000 g for 15 min, the supernatant was mixed in a 1:1 ratio with phenol (pH 8.0) (Thermo Fisher Scientific, USA), centrifuged at 12 000 g for 5 min. The upper phase was taken for precipitation of proteins. Five volumes of cold 100 mM ammonium acetate in methanol were added and incubated for 30 min at –20 °С. After centrifugation at 12 000 g for 5 min, the pellet was washed twice with 100 mM ammonium acetate in methanol and twice with 80 % acetone. The precipitate was dried in air and dissolved in the buffer for isoelectric focusing (25 mM tris-HCl (pH 8.0), 9 M urea, 4 % CHAPS, 50 mm DTT, 0.2 % ampholytes (Bio-Rad Laboratories, USA)). Protein concentration was measured using Bradford assay (Bradford, 1976).

Two-dimensional differential gel electrophoresis. Twodimensional differential gel electrophoresis (DIGE) of proteins was performed using staining of samples with various fluorescent dyes (Voss, Haberl, 2000). The samples were conjugated for 30 min on ice with fluorescent dyes Cyanine 2 or Cyanine 5 (Cy2 or Cy5) in various combinations. The incubation solution contained 400 pM of each dye dissolved in dimethylformamide for 30 min on ice. The reaction was stopped by adding 10 mM L-lysine (Sigma-Aldrich), followed by incubation on ice for 10 min. After that, the control and experimental samples were mixed, DTT and ampholytes (50 mM DTT, 0.2 % ampholytes (Bio-Rad Laboratories) were added. Passive in-gel rehydration with immobilized pH gradient (Bio-Rad Laboratories) was performed overnight at room temperature. The total amount of sample applied to 7 cm gel (pH 3–10, Bio-Rad Laboratories) was up to 100 μg. Isoelectric focusing (IEF) was performed in a Protean IEF system (Bio-Rad Laboratories) at a temperature of 20 °С, the samples were desalted at 250 V for 15 min, after which the voltage was linearly increased to 4,000 V for 2 hours, then IEF was carried out with increasing voltage up to 10 000 V. Before electrophoresis in polyacrylamide gel (PAGE), protein recovery was carried out in buffer with DTT (6 M urea, 0.375 M tris, pH 8.8, 2 % SDS, 20 % glycerol, 2 % DDT) for 10 min followed by alkylation in iodoacetamide buffer (6 M urea, 0.375 M tris, pH 8.8, 2 % SDS, 20 % glycerol, 2.5 % iodoacetamide) for 15 min. The second direction of two-dimensional electrophoresis was carried out in tris-glycine buffer (25 mM Tris-HCl, 192 mM glycine, 0.1 % SDS, pH 8.3) in 15 % polyacrylamide gel using a 4 % stacking gel. After separation of proteins the gels were visualized using a laser scanner Typhoon FLA 9500 (GE Healthcare, Germany).

Mass spectrometry. The proteins were rehydrated in trypsin solution (20 ng/μl trypsin, 30 mM tris, pH 8.2) on ice for 1 h and then incubated for 1 h at 56 °С. The peptides were extracted from the gel with 50 % acetonitrile, 0.1 % formic acid. This solution was evaporated in vacuum concentrator CentriVap (Labconco) at 4 °С and dissolved in phase A (5 % acetonitrile, 0.1 % formic acid). Mass spectrometry was performed using Agilent ESI-Q-TOF 6538 UHD (Agilent Technologies) combined with high performance liquid chromatograph Agilent 1260 (Agilent Technologies). Chromatography was performed in system water – acetonitrile in the presence of 0.1 % formic acid (phase A – 5 % acetonitrile with 0.1 % formic acid, phase B – 90 % acetonitrile with 0.1 % formic acid) in the gradient of acetonitrile (from 5 to 60 % phase B for 25 min and to 100 % phase B for 5 min) on Zorbax 300SBC18 column 3.5 μm, 150 mm length (Agilent Technologies) with flow rate 15 μl/min.

RNA extraction and quantitative reverse transcription PCR (RT-PCR). RNA extraction and RT-PCR were performed as described previously (Kirienko et al., 2018). The quantitative RT-PCR analysis was performed on a CFX-96 real-time PCR detection system with C1000 thermal cycler (Bio-Rad Laboratories). All primer pairs (Table 1) were designed using the Vector NTI program and produced by the Evrogen company (www.evrogen.com). PCR amplification specificity was verified using a dissociation curve (55–95 °С). mRNA levels were normalized against Ubiquitin and values were calculated as ratios relative to non-inoculated root expression levels. The data of two-three independent biological experiments were analysed. Statistical analysis was conducted by Student’s test ( p < 0.05) to assess the differences between variants.

**Table 1.**
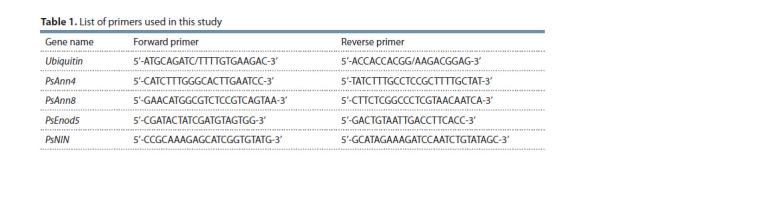
List of primers used in this study

Mass spectrometry. The proteins were rehydrated in trypsin solution (20 ng/μl trypsin, 30 mM tris, pH 8.2) on ice for 1 h and then incubated for 1 h at 56 °С. The peptides were extracted from the gel with 50 % acetonitrile, 0.1 % formic acid. This solution was evaporated in vacuum concentrator CentriVap (Labconco) at 4 °С and dissolved in phase A (5 % acetonitrile, 0.1 % formic acid). Mass spectrometry was performed using Agilent ESI-Q-TOF 6538 UHD (Agilent Technologies) combined with high performance liquid chromatograph Agilent 1260 (Agilent Technologies). Chromatography was performed in system water – acetonitrile in the presence of 0.1 % formic acid (phase A – 5 % acetonitrile with 0.1 % formic acid, phase B – 90 % acetonitrile with 0.1 % formic acid) in the gradient of acetonitrile (from 5 to 60 % phase B for 25 min and to 100 % phase B for 5 min) on Zorbax 300SBC18 column 3.5 μm, 150 mm length (Agilent Technologies) with flow rate 15 μl/min.

RNA extraction and quantitative reverse transcription PCR (RT-PCR). RNA extraction and RT-PCR were performed as described previously (Kirienko et al., 2018). The quantitative RT-PCR analysis was performed on a CFX-96 real-time PCR detection system with C1000 thermal cycler (Bio-Rad Laboratories). All primer pairs (Table 1) were designed using the Vector NTI program and produced by the Evrogen company (www.evrogen.com). PCR amplification specificity was verified using a dissociation curve (55–95 °С). mRNA levels were normalized against Ubiquitin and values were calculated as ratios relative to non-inoculated root expression levels. The data of two-three independent biological experiments were analysed. Statistical analysis was conducted by Student’s test ( p < 0.05) to assess the differences between variants.

Genetic constructs for plant transformation. To obtain the pBIN19 vector for plant transformation, carrying the gene of interest, the coding sequence of PsAnn4 gene without stopcodon has been amplified using cDNA as a template with corresponding primers (see Table 1). Total RNA was isolated from 2 dai pea roots of cv. Frisson. Amplification was done using Phusion Flash High-Fidelity PCR Master Mix (Thermo Scientific). The amplified products were restricted with XbaI and EcoRI and subcloned in the pMON vector under 35S promoter in the frame with the sequences encoding RFP or YFP and nopaline synthase terminator (Tnos). The inserts were verified by sequencing. The cassette composed of the 35S promoter, gene of interest fused with RFP or YFP and Tnos was excised from pMON using Hind III, SmaI and cloned in the pBIN19. All verified constructs were transferred into the Agrobacterium tumefaciens LBA4404.

Transient protein expression in N. benthamiana leaves. A. tumefaciens strain LBA4404 was used for infiltration in N. benthamiana leaves. Bacterial culture was grown at 28 °C overnight, then centrifuged at 3000 g and resuspended in 10 mM MES-KOH, 10 mM MgCl2 and 0.5 mM acetosyringone up to culture density OD600 = 0.5. Bacterial cells were infiltrated into the leaves of 3-week-old N. benthamiana. Plants were analyzed 48–96 h after infiltration.

Results

Phylogenetic analysis of annexins in pea and other legumes

The search of the sequences presumably coding for annexins in legumes was performed using BlastX with 8 previously revealed M. truncatula and 13 P. vulgaris nucleotide sequences encoding these proteins (Kodavali et al., 2013; Carrasco-Castilla et al., 2018) as queries against different plant sequence databases: https://phytozome.jgi.doe.gov/pz/ portal.html for M. truncatula and P. vulgaris, http://www.kazusa. or.jp/lotus/ for L. japonicus, and the URGI database v. 1 https://urgi.versailles.inra.fr/blast for P. sativum L. (Clark et al., 2001; Carrasco-Castilla et al., 2018; Kreplak et al., 2019). As a result, we were able to identify 18 coding sequences (CDSs) for annexins in M. truncatula, 15 in P. sativum L., and 13 in L. japonicus (Table 2). Twenty-three genes had been previously found to encode annexins in soybean (Feng et al., 2013). The coding sequences for annexins from P. sativum were named based on their phylogenetic relationships with the corresponding homologous sequences from M. truncatula and P. vulgaris (see Table 2) (Clark et al., 2012; Kodavali et al., 2013; Carrasco-Castilla et al., 2018).

**Table 2.**
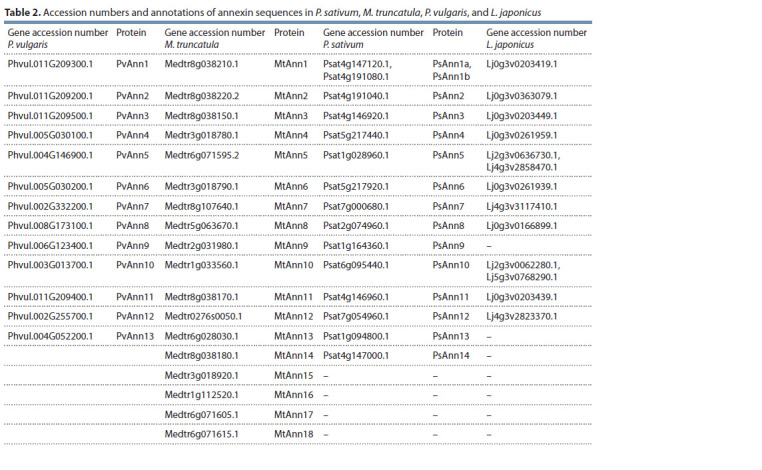
List of primers used in this study

**Fig. 1.**
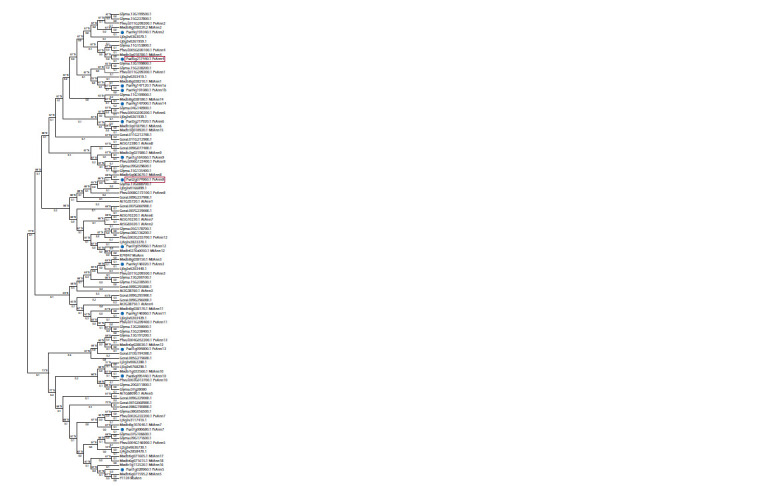
Phylogenetic tree of annexin sequences from legumes (P. sativum, G. max, M. truncatula, and P. vulgaris) and non-legumes (A. thaliana, G. barbadense, and G. hirsutum). The phylogenetic tree was generated with the maximum-likelihood method using MEGAX with 1,000 bootstrap replicates. PsAnn4 and PsAnn8 are indicated in boxes. The annexin sequences from P. sativum are indicated with blue circles

The phylogenetic analysis (Fig. 1) was performed using the deduced amino acid sequences of annexins found and annotated for P. sativum along with those of other legumes (M. truncatula, P. vulgaris, Lotus japonicus, and Glycine max) and non-legumes (A. thaliana, G. raimondii), which were available in the Phytozome database v. 12.1 and other databases.

Based on our analysis, the previously found MtAnn1 (Medtr8g038210) and PvAnn1 (Phvul.011g209300) clustered in the subclade with proteins corresponding to P. sativum Psat4g147120 and Psat4g191080, named PsAnn1a and PsAnn1b (see Table 2). Revealed in M. truncatula MtAnn2 (Medtr8g038220) and P. vulgaris PvAnn2 (Phvul.011g209200) clustered in the subclade with Psat4g191040, named PsAnn2.

Two previously described pea annexins induced in roots in response to rhizobial inoculation (Leppyanen et al., 2018) were identified as proteins corresponding to Psat5g217440 and Psat2g074960 coding sequences using a new database https://urgi.versailles.inra.fr/blast for P. sativum (see Table 2) (Kreplak et al., 2019). The phylogenetic analysis depicted an additional branch in the phylogenetic group with MtAnn1/ PvAnn1 and MtAnn2/PvAnn2, comprising MtAnn4 (Medtr3g018780), PvAnn4 (Phvul.005g030100), and their homolog Psat5g217440, named PsAnn4 (identified by proteomic screening) (see Table 2). Another previously found pea annexin, Psat2g074960, might be closely related to Medtr5g063670 and Phvul.008G173100.1, defined as MtAnn8 and PvAnn8 based on phylogenetic analysis (see Table 2).

Analysis of the domain composition of pea annexins and modeling of three-dimensional structure of PsAnn4 and PsAnn8

**Fig. 2.**
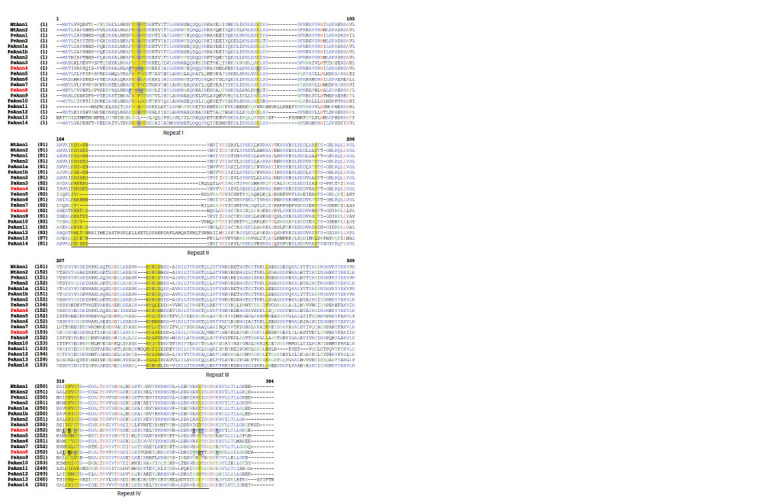
Multiple sequence alignment of the amino acid sequences of 15 presumable P. sativum annexins, 2 M. truncatula annexins (MtAnn1, MtAnn2), and 2 P. vulgaris annexins (PvAnn1, PvAnn2) by ClustalΩ. Four annexin repeats are underlined. Yellow highlights indicate potential calcium-binding motifs. In the calcium-binding motif of the first annexin repeat, the conservative tryptophan (W) necessary for binding to the membrane is indicated in gray. Important for calcium binding amino acid residues in the calcium-binding site of the type II (repeat I, Phe-23, Gly-25, Gly-27, and Glu-67) as well as in the calcium-binding site of the type III (repeat IV, Ile- 254, Lys-256, Gly-258, and Val-296, Thr-299, Glu-304) are indicated in bold and underlined. P. sativum annexins PsAnn4 and PsAnn8 are marked in red.

Analysis of the domain composition of the corresponding proteins in pea showed the presence of four typical domains of plant annexins (Fig. 2). This suggests that the annexin gene family indeed comprises several members in pea. Although plant annexins have four putative annexin repeats, not all Ca2+- binding motifs in these repeats seem to be functional. In plant annexins, the Ca2+-binding site is highly conservative in the first (I) repeat but is not conservative in the second (II) and third (III) repeats, while in the fourth (IV) repeat moderate conservatism is preserved (see Fig. 2).

**Fig. 3.**
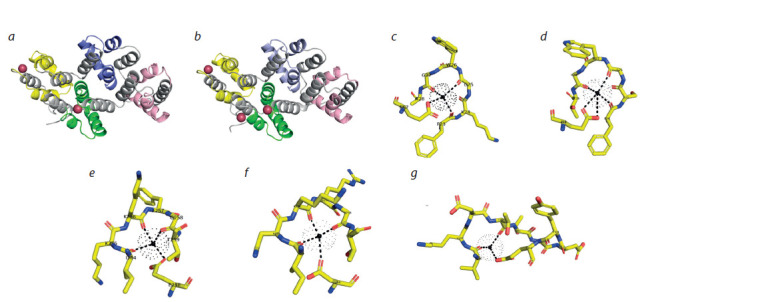
Modeling of the three-dimensional structures of PsAnn4 (a) and PsAnn8 (b) using the crystal structure of G. hirsutum annexin (GhAnn1, PDB code 3BRX) as a template and their binding with calcium ions in the f irst (c, d ) and fourth repeats (e, f, g). The 3D structures of PsAnn4 and PsAnn8 proteins indicated the coordination of calcium ions in the f irst (c, d ) and fourth (e, f, g) annexin repeats.

The crystal structure of the Gossypium hirsutum annexin GhAnn1 bound to calcium was obtained in an earlier study (Hu et al., 2008). Since PsAnn4 and PsAnn8 may be involved in regulation of pea-rhizobial symbiosis, we modeled the three-dimensional (3D) structure of these two annexins using GhAnn1, with 50 % sequence identity for PsAnn4 and 78 % sequence identity for PsAnn8 as a template (Fig. 3, a, b). The resulting 3D structures of PsAnn4 and PsAnn8 proteins indicated the coordination of calcium ions in the first and fourth annexin repeats. In the first repeat of both proteins, the calcium-binding site of the type II was coordinated by three carbonyl oxygen atoms of the residues Phe-23, Gly-25, and Gly-27, and carboxylate of Glu-67 in PsAnn4 and PsAnn8 (see Fig. 2 and 3, c, d ), as was shown earlier for GhAnn1 (Hu et al., 2008).

We suppose that the second calcium ion is bound in the loop of the fourth annexin repeat of PsAnn4 and PsAnn8 proteins. It is coordinated in the binding site of type II by Ile-254, Lys- 256, and Gly-258 in pea annexins (see Fig. 2, 3, e, f ). The third calcium ion (in the binding site of type III) is coordinated by two oxygen atoms of the residues Val-296 and Thr-299 and carboxylate of Glu-304 in this protein (similarly, Val, Thr, and Glu are involved in Ca2+ binding in the fourth repeat of GhAnn1) (see Fig. 2, 3, g) (Hu et al., 2008). However, in the fourth repeat of PsAnn4 protein, the Val-296 is replaced by Ser and Glu-304 by Lys (see Fig. 2). This might potentially obstruct the binding of the calcium ion, as was shown in our modeling (see Fig. 3, g). Although we cannot rule out that this might be due to low homology between PsAnn4 and GhAnn1, which was used as a template in the modeling, the results suggest the potential difference in Ca2+ binding between PsAnn4 and PsAnn8 proteins.

Comparative analysis of protein patterns in wild-type and non-nodulating pea mutant

To verify whether the stimulation of synthesis of PsAnn4 and PsAnn8 proteins depends on Nod factor perception, the protein patterns were analyzed in wild-type pea cv. Frisson and a P56 mutant with a defective sym10 gene (which encodes a putative Nod factor receptor) (Madsen et al., 2003).

**Fig. 4.**
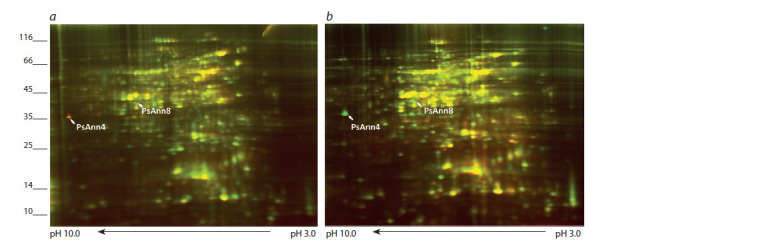
Comparative analysis of protein patterns in wild-type pea plant and P56 mutant with an impaired sym10 gene using two-dimensional differential gel electrophoresis 1 day after inoculation (1 dai). The protein extract from wild type pea roots inoculated with R. leguminosarum bv. viciae RCAM1026 was labelled with Cy2 (red) and protein extract from inoculated roots of P56 mutant was labelled with Cy5 (green) (a) and conversely the extract from inoculated wild type roots was labelled with Cy5 (green) and protein extract from inoculated roots of P56 mutant was labelled with Cy2 (red) (b).

Two-dimensional differential in-gel electrophoresis-based proteomics was used to characterize the pattern of protein distribution (Fig. 4). Two spots corresponding to the location of the previously characterized annexins (Leppyanen et al., 2018) were excised from the gel. Mass spectrometric analysis confirmed their identity to annexins Psat5g217440 (PsAnn4) and Psat2g074960 (PsAnn8). Enhanced level of PsAnn4 was found in the inoculated roots of wild type pea plants (cv. Frisson) compared to the inoculated P56 mutant roots.

The amount of PsAnn8 protein was also slightly higher in response to inoculation in the wild type than in the P56 mutant, but not as essential as for PsAnn4. In accordance with this, low amounts of PsAnn4 and PsAnn8 proteins were found in the roots of the P56 mutant and didn’t change in response to inoculation. This suggests that the up-regulation of both annexins may depend on Nod factor recognition in pea plants and may be connected with the functioning of these annexin during symbiotic interaction of plants with rhizobia at early stages. Since the increase in the amount of PsAnn4 protein was more significant in response to inoculation, we focused on this annexin in our next experiments

PsAnn4 expression pattern in response to rhizobial inoculation and treatment with Ca2+ inhibitors

**Fig. 5.**
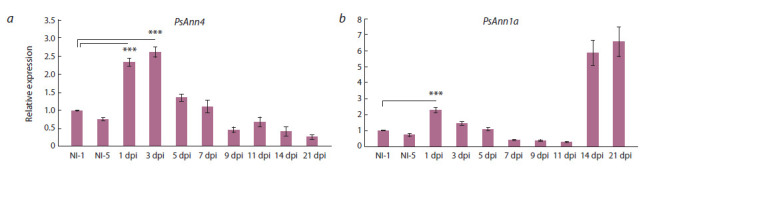
Quantitative RT-PCR analysis of PsAnn4 (a) and PsAnn1b (b) expression in pea roots upon nodulation. mRNA levels were normalized against Ubiquitin and values were calculated as ratios relative to non-inoculated root (NI) expression levels. The data of three independent biological experiments were analyzed. Bars represent the mean ± SEM of two biological replicates. Asterisks indicate signif icant differences compared to non-inoculated roots, based on Student’s t-test and p-value less than 0.001 is f lagged with three asterisks (***).

The PsAnn4 expression pattern in response to rhizobial inoculation was analyzed in our experiments (Fig. 5, a). A quantitative RT-PCR analysis revealed that Rhizobium infection enhanced the PsAnn4 gene expression at the early stages of nodulation, starting from 1–3 days after inoculation up to 5 days after inoculation, but thereafter their transcript levels did not significantly change upon nodule development (see Fig. 5, a). In our experiments the expression of another annexin gene, PsAnn1a, the closest homolog of MtAnn1 gene was also analyzed (see Fig. 5, b). As it was expected, the PsAnn1a gene expression was primarily enhanced at the early stages of symbiosis development and reached the highest levels in the nodules. Similar pattern had been previously found for MtAnn1 (De Carvalho-Niebel et al., 1998, 2002). Therefore, up-regulation of PsAnn4 expression may be related to the early stages of nodulation. The upregulation of the PsAnn4 transcription level was not as significant as it was at the protein level, which implies that the regulation of this annexin can be mainly achieved at the post-transcriptional and translational level.

**Fig. 6.**
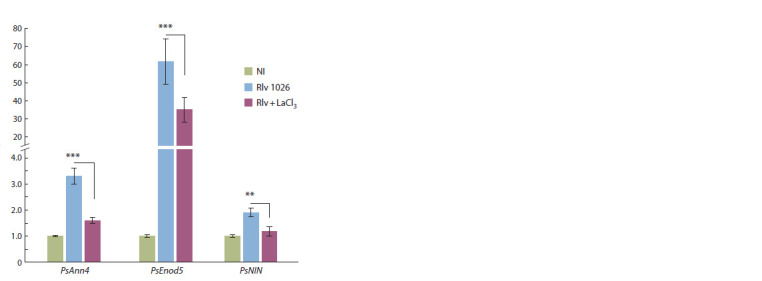
PsAnn4, PsEnod5, and PsNIN expression levels in pea roots after inoculation (1 dai) with R. leguminosarum bv. viciae RCAM1026 (Rlv) and after treatment with the Ca2+ channel blocker LaCl3 (Rlv + LaCl3). mRNA levels were normalized against Ubiquitin and values were calculated as ratios relative to non-inoculated root expression levels (NI). The data of three independent biological experiments were analyzed. Bars represent the mean ± SEM. Asterisks indicate signif icant differences between treated (Rlv + LaCl3) and non-treated (Rlv) roots, based on Student’s t-test and p-values less than 0.001 and 0.01 are f lagged with three (***) and two (**) asterisks, respectively.

To verify the influence of calcium inhibitors on the regulation of PsAnn4 gene, its expression level was estimated after plant treatment with the Ca2+ channel blocker LaCl3 (Fig. 6). Two previously described as symbiosis-specific genes PsNIN and PsEnod5 were also used in our experiments as a control for effective inoculation. In pea roots, the upregulation of PsAnn4 expression in response to inoculation was revealed in 1 dai, corresponding with experiments on the dynamics of this gene expression upon nodulation. The significant decrease in the expression of PsAnn4 was found in our experiments in the presence of LaCl3. Down-regulation of symbiosis-specific genes PsEnod5 and PsNIN was also observed, which indicated the importance of Ca2+ influx for their regulation. Therefore, the influx of calcium ions into the cell, which is observed at the early stages of symbiosis development, may affect the expression level of PsAnn4 in pea roots (see Fig. 6).

Subcellular localization of pea PsAnn4 annexin

**Fig. 7.**
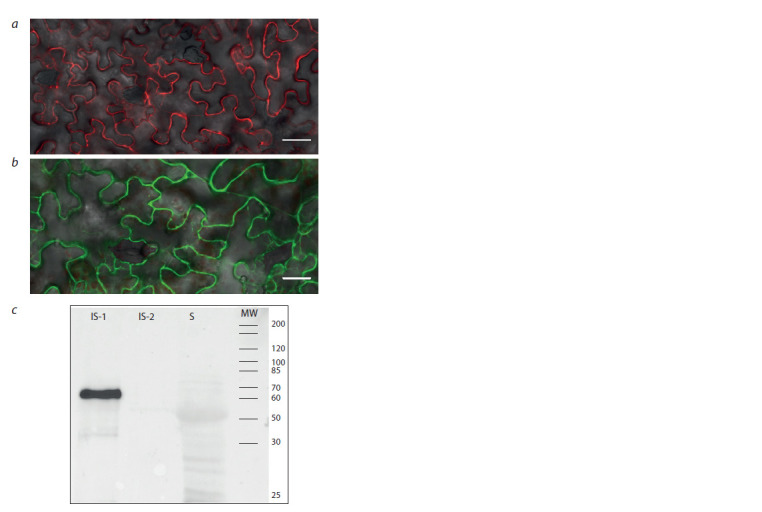
Localization of PsAnn4 fused to red f luorescent protein (RFP) (a) and yellow f luorescent protein (YFP) (b) at the C-terminus under the transcriptional regulation of the 35S promoter in N. benthamiana leaves by in- f iltration with A. tumefaciens LBA4404. Scale bars are 200 μm. Immunoblot analysis of different cell fractions obtained from the N. benthamiana leaves after inf iltration of PsAnn4-YFP with A. tumefaciens LBA4404 (c). IS-1 – insoluble fraction was pelleted at 36 000 g; IS-2 – insoluble fraction was pelleted at 100 000 g; S – soluble fraction at 100 000 g; MW – molecular weight marker.

To follow the PsAnn4 protein localization in plant cells, it was fused to the fluorophores such as red fluorescent protein (RFP) and yellow fluorescent protein (YFP) at the C-terminus and expressed under the transcriptional regulation of the 35S promoter in N. benthamiana leaves by infiltration with A. tumefaciens (Fig. 7, a, b). The infiltration of constructs for the synthesis of proteins fused with RFP and YFP allowed us to visualize the protein in leaf tissues after transformation. In the cells of N. benthamiana leaves, PsAnn4 protein was localized in the plasma membrane or in the cell wall. In addition, we also estimated the presence of PsAnn4 in different cell fractions by Western-blot hybridization using anti-YFP or anti-RFP antibodies. PsAnn4-YFP was found in insoluble fraction of leaf tissue pelleted at 36 000 g (see Fig. 7, c). It suggests that PsAnn4 may be involved in cell wall or membrane modification as well as in ion transport.

Discussion

Available pea genome information (Kreplak et al., 2019) allowed us to determine the composition of the annexin gene family in this legume. Database searches revealed 15 annexin genes in P. sativum L., 18 in M. truncatula as well as 13 in both P. vulgaris and L. japonicus. Based on the phylogenetic analysis of these annexins, close homologs can be identified among these legume species (see Fig. 1).

At present, only one pea annexin, p35, has been functionally characterized (Clark et al., 1992). The localization of this annexin in root cells involved in active secretion suggests its function in exocytosis. Subsequently, the use of antibodies against this protein revealed its localization in epidermal cells of the leaf and stem (Clark et al., 1998, 2000). However, annexins involved in nodulation have not been characterized in P. sativum. In contrast, in M. truncatula, two annexins, MtAnn1 (Medtr8g038210) and MtAnn2 (Medtr8g038220),demonstrated a high level of expression during nodulation and were found to be involved in controlling bacterial infection and nodule organogenesis (De Carvalho-Niebel et al., 1998, 2002; Manthey et al., 2004; Breakspear et al., 2014). Another annexin, MtAnn3 (Medtr4g097180), was found to be important for root hair deformations in M. truncatula (Gong et al., 2012). At the same time, close homologs of MtAnn1 – PvAnn1 (Phvul.011g209300) and LjAnn1 (Lj0g3v0203419), which belong to the same phylogenetic group as MtAnn1, play important roles in the symbiotic process in P. vulgaris and L. japonicus (Wienkoop, Saalbach, 2003; Jáuregui-Zúñiga et al., 2016; Carrasco-Castilla et al., 2018).

In our earlier work, two annexins activated at the early stages of symbiosis development in pea were found using the proteomics approach (Leppyanen et al., 2018). This approach might be helpful for the identification of new regulators of signal transduction pathways at the initial stages of nodulation in pea. Our present analysis revealed that these two identified annexins of pea belong to different phylogenetic groups, defined as homologs of MtAnn4, PvAnn4 and MtAnn8, PvAnn8, respectively. Although PsAnn4, and MtAnn4 and PvAnn4 have high levels of homology with MtAnn1 and PvAnn1, they belong to another group of annexins based on phylogenetic analysis. PsAnn8 belongs to a less studied phylogenetic group. Therefore, two previously unknown annexins were identified in our study. In addition to stimulation during rhizobial inoculation, the dependence of PsAnn4 and PsAnn8 activation on the LysM-receptor-like kinase SYM10, encoding a putative Nod factor receptor, was revealed in the present study (see Fig. 4), which suggested that rhizobial signaling molecules Nod factors may be important for their activation. It also suggests the participation of these two annexins in the development of the symbiotic interaction of plants with rhizobia.

Phylogenetic analysis and prediction of the overall 3D structure of PsAnn4 and PsAnn8 proteins showed differences in the Ca2+-binding motif in the fourth annexin repeat of these proteins, and therefore, in the potential ability to bind calcium ions. This can potentially influence the binding of these annexins to phospholipids by means of a calcium bridge mechanism. It was predicted that three calcium ions were coordinated in the first and fourth repeats, which is consistent with the data of the canonical binding of the G. hirsutum annexin GhAnn1 and animal annexins to the phospholipids of membranes using the mechanism of calcium bridges (Hu et al., 2008). In the predicted structures of Arabidopsis annexins (AtAnn1, AtAnn3, and AtAnn4), the canonicity of the Ca2+-binding motif in the first repeat and the presence of modified motifs in the fourth repeats of AtAnn1 and AtAnn3 were also shown, while AtAnn4 had no recognizable Ca2+ – or phospholipidbinding motifs (Konopka-Postupolska, Clark, 2017).

Since the level of PsAnn4 synthesis in response to inoculation was more significant in the roots of wild type pea plants compared with mutant defective in symbiosis, we carried out the analysis of this annexin in more detail. It was shown that the regulation of PsAnn4 annexin in pea could be achieved at the transcriptional level as well as post-transcriptional and translational levels, probably. Significant activation of MtAnn1 and MtAnn2 gene expression level was found in the roots of M. truncatula treated with Nod factors or inoculated with rhizobia (De Carvalho-Niebel et al., 1998, 2002; Manthey et al., 2004; Breakspear et al., 2014). Meanwhile, the expression of PvAnn1 in P. vulgaris was slightly upregulated in developing nodules (Carrasco-Castilla et al., 2018). However, a phosphoproteomic approach revealed that PvAnn1 was a phosphorylated protein with enhanced levels of synthesis during nodulation (Jáuregui-Zúñiga et al., 2016). Hence, the regulation of annexins involved in nodulation might be different and is probably connected with different functions that annexins fulfil in this process

Localization of annexins might differ depending on their function. Some annexins show cytoplasmic and nuclear localization, while other annexins are associated with various plant membranes, including the plasma membrane, endoplasmic reticulum, and nuclear membrane (Laohavisit, Davies, 2011; Clark et al., 2012; Davies, 2014). Some annexins may be embedded in the membrane in the form of monomers or oligomers. One of the distinctive characteristics of annexins is their ability to change their cellular localization in response to various stimuli. In our experiments, the localization of annexin 4 (PsAnn4) in the cell wall or plasma membrane was shown, suggesting the participation of this annexin in processes associated either with membrane modification or ion transport at the early stages of symbiosis establishment in pea. Similarly, the localization of the other annexin, MtAnn2, involved in nodulation in M. truncatula, was revealed to be associated with the plasma membrane, particularly with lipid rafts from root plasma membrane preparations (Lefebvre et al., 2007). In addition, the annexin PvAnn1 is essential for ROS-dependent regulation of Ca2+ influx into the cells of P. vulgaris, which strongly suggests the localization of this protein in the plasma membrane. Therefore, specific subcellular localization of annexins might be associated with their function signal transduction at the early stages of symbiosis.

## Conclusion

In this study, phylogenetic analysis of the pea annexins PsAnn4 and PsAnn8 was performed based on their homology with annexins from other legumes. The modeling approach allowed us to estimate the structural features of these annexins that might influence their functional activity. To verify the functions of these annexins, we performed comparative proteomic analysis, experiments with calcium influx inhibitors, and localization of labeled proteins. Essential down-regulation of PsAnn4 synthesis in a non-nodulating pea mutant P56 (sym10) suggests an involvement of this annexin in the rhizobial symbiosis. The localization of PsAnn4 in the cell wall or plasma membrane of plant cells may indicate its participation in membrane modification or ion transport.

## Conflict of interest

The authors declare no conflict of interest.
